# Editorial: Rising stars in integrative neuroscience 2021

**DOI:** 10.3389/fnint.2023.1229660

**Published:** 2023-06-14

**Authors:** Giuseppina Porciello, Lucia Maria Sacheli

**Affiliations:** ^1^Department of Psychology, Sapienza University of Rome, Rome, Italy; ^2^Istituto di Ricovero e Cura a Carattere Scientifico (IRCCS) Santa Lucia Foundation Hospital, Rome, Italy; ^3^Department of Psychology and Milan Center for Neuroscience, University of Milano-Bicocca, Milan, Italy

**Keywords:** social interaction, developmental neuroscience, social touch, social decision making, brain-immune network, non-invasive brain stimulation

The Rising stars in Frontiers in integrative neuroscience 2021 aims to gather ideas proposed by internationally recognized researchers in the early stages of their careers. We asked them to delineate the forthcoming pathways for integrative neuroscience by portraying theories and methodologies that, according to them, are likely to impact the field, particularly human cognitive neuroscience research. The contributions provide diverse perspectives to enhance our understanding of the role of neurofunctional brain networks in integrating multiple functions to generate meaningful, adaptive, and social behaviors.

Three of these contributions delve into the astonishing human ability to navigate the social world. Curioni explores when and why humans prefer to act together rather than alone to achieve a certain goal. The author investigates whether this preference is rooted in an intuitive utility calculus that considers the costs (e.g., anticipated effort) and benefits (e.g., potential reward) associated with individual and joint action alternatives. *Why do we act together even when it is not necessary or instrumentally beneficial?* The article suggests that people's decision-making in this regard becomes reasonable and predictable only when we assume interactions are intrinsically rewarding, driving people to establish social relationships. Understanding how this rewarding value is computed at the neurophysiological level and identifying factors that modulate this computation is one of the challenges that social neuroscience research should face in the future.

Feruglio et al. examine the social impact of mindfulness meditation, a practice that trains individuals to adopt an open, gentle, and self-compassionate mindset while focusing on their internal experiences. Although this technique primarily focuses on inner and subjective states, it appears to strongly affect social and moral attitudes toward others. Again, the reward system seems to play a role but current empirical approaches suffer from methodological flaws. Indeed, since mindfulness improves self-control and reduces reward salience, the authors raise the question: *Does mindfulness induce people to give money to others for their sake's wellbeing or does it reduce the attracting power of money, making it easier to give it away?* The authors call for a multidisciplinary and integrative approach to unveil the underlying mechanisms while preventing interpretation biases.

Fusaro et al. instead review the evidence supporting the idea that others' caresses can be proposed as a novel treatment to reduce the subjective feeling of pain, particularly in clinical populations suffering from chronic pain (CP). Pleasant touch plays a crucial role in social interactions, and it is beneficial to healthy individuals' wellbeing in various stressful situations. However, the underlying mechanisms of this beneficial effect and possible clinical applications are not yet clear. The authors suggest that the efficacy of touch-based therapies in people with CP may be influenced by their alteration in the perception of tactile pleasantness (tactile allodynia). Immersive Virtual Reality (IVR) paradigms may be used to overcome this limitation, as they allow people to experience pleasant vicarious touch-related sensations without actual physical stroking. These techniques could be applied to restore the positive value of pleasant touch and develop successful treatments for CP. In this contribution emerges the usefulness that IVR techniques may play in clinical settings in the future and the major role that a social experience like affective touch may play in shaping the intimate experience of feeling pain.

More general and theoretical contributions are those provided by Wass and Goupil and Ciaunica et al.. The former discusses current methodological limitations and novel promising approaches to investigate how the brain, particularly the developing brain, interacts with a dynamic real-world environment. The authors argue that the current reductionism in cognitive neuroscience infant research may not be ideal for understanding how cognitive operations unfold outside the laboratory and suggest fostering solutions embracing the complexity of real-world situations. These solutions include the application of data-driven approaches to explore participants' spontaneous responses to naturally/pseudo-naturally occurring environmental changes, measuring the bidirectional relationship between fluctuating brain states and individual/dyadic behaviors, as well as conducting cross-linguistic and cross-cultural studies. These approaches enable a better consideration of the active role of the (developing) individual in shaping perceptual and cognitive processing.

By reviewing recent and past evidence around the question that the human mind is separate from the physical body and has a special connection to the brain, Ciaunica et al. propose that shifting the focus from neural to cellular processing may provide new insights for improving our understanding of human cognition, and challenge the notion that mental states are uniquely associated with the brain. The authors argued that although neurons are essential for neural processing and cognitive functions, other types of cells, such as immune cells, nowadays neglected in neuroscientific research, also support cognitive processes and play a crucial role in maintaining the self-organization of the human body. By describing the multiple interactions between the nervous and immune systems Ciaunica et al. suggest that studying the “brain-immune” network may open new venues for characterizing the complexity of human cognition.

Finally, embracing a more methodological perspective, Fusco et al. discuss the potential of the causal approach allowed by non-invasive brain stimulation (NIBS) techniques in elucidating the neurophysiological mechanisms that underpin brain-behavior relationships. The authors use performance monitoring as an example of a crucial, adaptive, and highly sophisticated competence that can be modulated by using NIBS. They also discuss methodological limitations and strategies to improve these techniques in the future, along with their potential clinical relevance. For example, what, where, and through which mechanisms we are currently neuromodulating remains unclear, and many factors like inter-subject variability, neural state dependency, and blinding procedures may all play confounding effects. Therefore, future improvements are necessary to reach highly rigorous standards.

In conclusion, the Rising stars in Frontiers in integrative neuroscience 2021 brought together ideas spanning from fundamental research to practical applications in both healthy individuals and clinical populations, from experimental approaches to theoretical frameworks, and from laboratory settings to ecological contexts. This collection encourages a comprehensive and holistic perspective in the study of the complexities of the human mind.

## Author contributions

All authors listed have made a substantial, direct, and intellectual contribution to the work and approved it for publication.

